# Microglia Phenotypes Converge in Aging and Neurodegenerative Disease

**DOI:** 10.3389/fneur.2021.660720

**Published:** 2021-05-05

**Authors:** Michael Candlish, Jasmin K. Hefendehl

**Affiliations:** Institute of Cell Biology and Neuroscience, Buchmann Institute for Molecular Life Sciences, Goethe University Frankfurt, Frankfurt am Main, Germany

**Keywords:** microglia, aging, neurodegeneration, alzheimer's disease, senescence

## Abstract

Microglia, the primary immune cells of the central nervous system, hold a multitude of tasks in order to ensure brain homeostasis and are one of the best predictors of biological age on a cellular level. We and others have shown that these long-lived cells undergo an aging process that impedes their ability to perform some of the most vital homeostatic functions such as immune surveillance, acute injury response, and clearance of debris. Microglia have been described as gradually transitioning from a homeostatic state to an activated state in response to various insults, as well as aging. However, microglia show diverse responses to presented stimuli in the form of acute injury or chronic disease. This complexity is potentially further compounded by the distinct alterations that globally occur in the aging process. In this review, we discuss factors that may contribute to microglial aging, as well as transcriptional microglia alterations that occur in old age. We then compare these distinct phenotypic changes with microglial phenotype in neurodegenerative disease.

## Introduction

**Figure 1:**
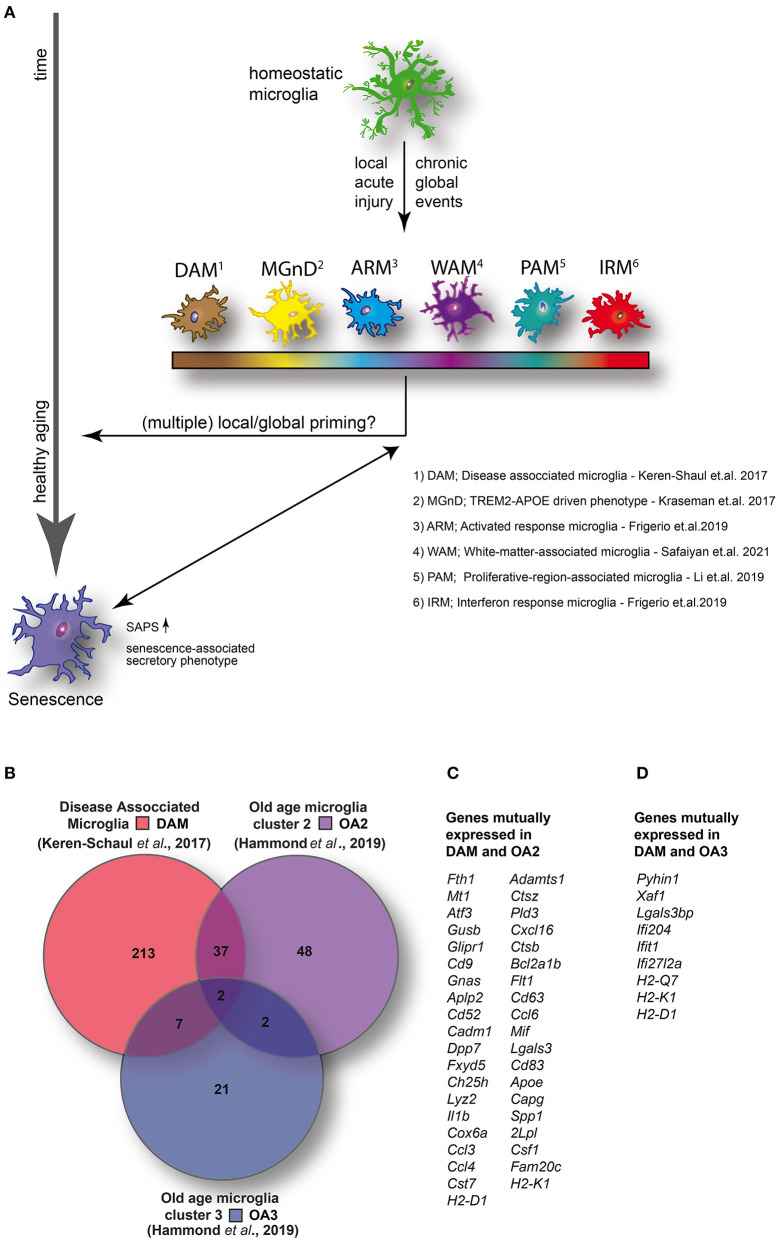
**(A)** Homeostatic microglia over the life span of the animal encounter both spatially restricted and global injuries that likely contribute to transcriptomic and functional alterations in microglia, potentially resulting in the adaption of specific disease- or injury-associated microglia phenotypes and conceivably contributing to premature cellular aging. **(B)** Venn diagram illustrating the number of enriched (1.5-fold or higher) mutually and exclusively expressed genes between DAM (identified in a mouse model of AD) (14) and two distinct clusters identified in aged wild-type mice [old age (OA) 2 and OA3] (7). **(C)** DAM and OA2 feature 39 mutually expressed genes suggesting the activation of common transcriptional programs in both neurodegenerative disease and a minority of cells in healthy aging. **(D)** Conversely, only nine genes were mutually expressed between DAM and OA3 suggesting that the OA3 population may be distinct from both DAM and OA2 microglia. Venn diagram generated using Venny 2.1 (https://bioinfogp.cnb.csic.es/tools/venny/).

Microglia originate from hematopoietic progenitor cells found in the yolk sac and, upon entering the brain, gradually adapt a homeostatic microglial phenotype (1). Homeostatic microglia feature a distinct ramified morphology and were first identified by del Rio-Hortega (2). As the primary immune cells of the brain, microglia are mostly associated with acute or chronic responses to injury. In response to these stimuli, microglia display morphological and biochemical changes that have often been grouped under the term “activation.” These changes can entail a variety of downstream effects including cytokine and chemokine production, enhanced phagocytosis, proliferation, and migration. Historically, based on *in vitro* experiments, this rather generalized diversion from the homeostatic cell state has led to a differentiation into two microglial groups: M1 (proinflammatory) and M2 (neuroprotective). This relatively oversimplified classification (3) of microglial reactivity is now being refined by single-cell resolution techniques that show diverse transcriptional states that can be adapted by microglia in either a gradual or acute manner. Intriguingly, we and others have shown that microglia are long-lived cells that undergo an aging process on a cellular level, altering their surveillance capacity and injury response time, and also influence neurodegenerative diseases (4–7) [reviewed in (8, 9)]. These rather slow and gradual alterations are contrasted by rapid changes brought on by acute damage. Local signals in the microglial microenvironment drive acute as well as gradual changes, leading to broad alterations in gene transcription, cell morphology, phagocytotic activity, and proliferation status (10–12). Over decades of research, a wide variety of terms have been used to describe microglial cell states (13). As research has advanced, new distinctions in microglial phenotypes have been identified based on expression of particular genes and, most recently, their transcriptome signature. In this review, we aim to uncover potential similarities in microglial phenotypes in advanced age and neurodegenerative disease (Figure 1A) (12, 15, 16).

## Microglial Aging: A Summation of Factors Accumulated Throughout Life?

It recently was shown that not only cells of the adaptive immune system but also those of the innate immune system can display memory effects (5). The priming step of the immune cells, induced by a primary insult, can result in an enhanced or dampened subsequent injury response (17–19). *In vivo* tracking of individual microglial cells showed that microglia can revert to a homeostatic morphology post-injury (20–22). However, even if the morphological homeostatic phenotype is reestablished, epigenetic modification may render the cells altered from their homeostatic state. As long-lived cells, microglia are very likely to be primed by signals in their microenvironment, hence memory effects might add up during the cell's life span. This poses the question if these rather individualized priming steps might be part of the cellular aging process or if they have the potential of adversely affecting healthy aging. Furthermore, as some acute injuries such as microlesions are likely to only affect surrounding microglial cells, the priming impact might be locally restricted.

Individual local events could result in the generation of distinct spatially restricted transcriptional and phenotypic alteration as microglial phenotypes are partially regulated through membrane-bound pattern recognition receptors (PRRs), which depend on molecules released by cells in their microenvironment. In a local ischemic event, disease-associated molecular patterns are passively released from dying cells (23), which may lead to a transient activation with a priming effect, whereas the deposition of amyloid β (Aβ) in the parenchyma [a hallmark of Alzheimer disease (AD), but can also be found in aged brains on a lesser scale] may lead to a different chronic disease–associated microglial phenotype, which not only depends on PRRs, as microglia also possess a wide variety of receptors to detect other types of molecules such as hormones and neurotransmitters (23). Therefore, a multitude of factors exist that could potentially affect local and global microglial behavior both in a short- or long-term fashion. A recent study demonstrated the importance of the local milieu within the brain in this regard by transiently depleting microglia using the colony-stimulating factor 1 receptor antagonist PLX5622 (24) in aged mice (25). The authors hypothesized that withdrawal of the drug would result in the replenishment of “young” unprimed microglia (25). Conversely, they reported that the transcriptomic alterations in old age were only partially reversed, and the replenished microglia responded to lipopolysaccharide (LPS) with an exaggerated proinflammatory response, typical of primed microglia. Further *in vitro* experiments confirmed that media conditioned by 24-h cultivation of brain slices from aged, but not young adult, mice were sufficient to trigger an exacerbated response to LPS in neonatal microglia, elegantly demonstrating the importance of the milieu in which microglia are resident (24).

When focusing on the healthy aging process of microglia, they have been described as dystrophic or senescent [reviewed in (26)]. Historically, senescence is characterized by arrested growth caused by oxidative stress as well as elevated DNA damage. Age-related changes in the secretory profile were described to coin the term *senescence-associated secretory phenotype*, classifying a particular cell state in the aging brain (27, 28). The term *dystrophic*, on the other hand, was created by the observation of changes in microglial morphology in brain sections from elderly humans and potentially includes all visually altered microglia (20). Among other features, this phenotype includes the beading of microglial processes, which are held together by thin channels (29), and was proposed to signify microglial senescence (30). Previous studies addressing the question if one of the described phenotypes is purely age-related are controversial; some have found dystrophic microglia in aged humans without any underlying neurodegenerative disorders (30–32), whereas others gathered evidence suggesting that dystrophic microglia are associated with a variety of diseases including, e.g., AD (29, 31–34), Huntington disease (14), and multiple sclerosis (35). However, recently some light has been shed on the question whether these two terms are describing the same or two different phenotypes. To address this issue, Shahidehpour et al. (36) conducted stereological analysis of microglia in human brain tissue spanning the age of 10-90 years. The analysis revealed an increased number of dystrophic microglia with age, which, however, was much greater when neurodegenerative pathology was present as well (36). They hence conclude that aging itself is only associated with a minor increase in dystrophic microglia (36). It is thus possible that the disease event that generated an activated microglia phenotype, potentially early in life, has a priming effect on cellular aging, leading to an increase of dystrophic microglia in old age (Figure 1A).

Also, the opposite assumption is valid; the overall cellular aging process is likely causative for poor local injury responses, resulting in an ineffective healing process that in turn might again increase the amount of dystrophic/senescent microglia. One example is the finding that population RNAseq of murine microglia has identified a consistent age-dependent increase in genes associated with a low-grade inflammatory response (37), which might be causative for a poor local injury response by microglia to additional acute insults. We have found the injury response time to a local laser lesion of aged microglia (~2.4-year-old mice) to be reduced by ~50%. Additionally, while microglial process end-tips in young and adult mice showed an increase in local diameters after a microlaser lesion, the aged animals displayed significantly less morphological changes upon lesioning, as the process end-tips already were found to be enlarged prior to the insult (4). Further supporting a gradual overall drift into a low-grade inflammatory state during aging, Minhas et al. recently put forward evidence suggesting that a change in the metabolic state of macrophages (in the brain and periphery), signified by a reduction of the two main metabolic pathways (glycolysis and oxidative phosphorylation), is affecting brain health as acute energy demands, e.g., in order to support macrophage activation, cannot be met any longer (38). More specifically, prostaglandin E_2_ (PGE_2_), a proinflammatory signaling protein, which is known to increase not only during aging but also in AD, was investigated. The inhibition of PGE_2_ was shown to lead to brain rejuvenation, reducing inflammatory levels in the aged brain (38). These findings are of particular interest as they support other reports that microglia can be influenced by stimulation of peripheral immune cells (5) and that their responsiveness can be (partially) restored even in the aged brain. With the detailed mechanism leading to the reported reduction in metabolism still being unknown, a future challenge is to unravel this pathway to explore possible therapeutic interventions, aiding in a wide range of diseases, as well as aging itself.

## Transcriptomic Alterations in Microglia in Old Age and Neurodegenerative Disease

In the effort to characterize microglia phenotype in disease, scRNAseq has become a powerful weapon and has facilitated even greater insight into the transcriptomic alterations of microglia in diverse conditions. At present, however, comparing transcriptomic data from different studies comes with inherent challenges. Variability can be introduced at many stages such as dissociation, gating for cell sorting, the scRNAseq procedure, and data analysis. Technical limitations can further result in discrepant data. For example, it was recently revealed that the technical limitations of single-nucleus (sn)RNAseq (a theoretically useful approach for postmortem human tissue as it is compatible with frozen tissue) may have resulted in data that lead inadvertently to the overstating of differences between murine and human microglial transcriptomes (16). Thrupp et al. found that many transcripts associated with microglial activation are concentrated in the cytosol as opposed to the nuclei, resulting in many transcripts remaining undetected with snRNAseq (16). Another pertinent issue is that despite recent technical advances in scRNAseq, correlating transcriptional profiles to mechanistic data remains a persistent bottleneck. Spatially resolved scRNAseq, especially at a single-cell resolution, would provide an unparalleled advantage in correlating morphological observations to distinct transcriptome signatures. At present, however, these limitations greatly hinder correlating microglial phenotypes (as evident by morphology or behavior *in vivo*) with specific gene-expression profiles.

Despite these difficulties, highly valuable data have been collected in several high-impact studies. Hammond et al. (12) identified two distinct microglia clusters using scRNAseq that, in the absence of overt pathology, were expanded in aged (~1.5 years old) mice. One cluster (entitled OA2) was found to up-regulate the chemokines *Ccl3* and *Ccl4* along with interleukin 1 beta (*Il1b*), indicative of a shift to a proinflammatory phenotype during aging (12). The other emerging cluster in aged animals was found to be enriched in several interferon-response genes [*Ifitm3, Rtp4*, and *Oasl2* (entitled OA3)] (12). This shift toward expression of interferon-response genes is highly interesting, given that recent research has demonstrated that interferon signaling in a mouse model of AD triggers microglial activation, neuroinflammation, and synaptic loss in response to nucleic acid containing Aβ plaques (39).

Sala Frigerio et al. (40) also identified two microglial clusters using scRNAseq that were expanded in aged mice. One cluster, entitled activated response microglia (ARM), increased from around ~3% in 3-month-old mice to ~12% of total microglia in 21-month-old mice (40). ARM microglia were found to up-regulate histocompatibility complex class II genes (*Cd74, H2-Ab1*, and *H2-Aa*) and proinflammatory genes *Cst7, Clec7a*, and *Itgax* (encoding CD11c). Notably, with the exception of *Itgax* and *Clec7a*, these genes were also found to be up-regulated in OA2 microglia (12). Another microglia cluster was also identified, dubbed interferon-response microglia (IRM) (40). This cluster was found to up-regulate *Ifit3, Ifitm3, Irf7*, and *Oasl2*, consistent with OA3 microglia (12). Furthermore, using semisupervised pseudotime analysis, the authors found that homeostatic microglia in old age can transition into either IRM or ARM. Taken together, these data suggest that in old age, microglia transition into one of two mutually exclusive states, one characterized by up-regulation of interferon-response genes and the other characterized by a shift to a proinflammatory state. However, as previously mentioned, it remains challenging to ascertain the impact that transcriptional alterations have on microglial phenotype *in vivo*.

Microglia play complex roles in neurodegenerative disease (41), often being beneficial in some respects, while pathogenic in others. Taking AD as an example, microglia phagocytose Aβ (42, 43) (although this becomes attenuated with aging/Aβ plaque load) and encircle Aβ plaques, prohibiting the spread of [comparably more toxic (44)] soluble amyloid species into the surrounding brain parenchyma (45). Consistent with this, ablation of microglia after amyloid deposition results in increased LAMP1 immunoreactivity surrounding Aβ plaques (46); indicative of dystrophic neurites (47). While these findings suggest that microglia play a beneficial role in AD, microglia themselves contribute to synaptic and neuronal loss in AD (48). It was also shown that abolishing microglia after Aβ plaques are well-established in the brain fails to provide any beneficial effects (49) in the APPPS1 AD mouse model. Furthermore, if microglia are abolished prior to amyloid deposition, Aβ plaques fail to develop in the 5XFAD AD mouse model (46) (although Aβ accumulates on blood vessels resulting in cerebral amyloid angiopathy, a risk factor for hemorrhagic stroke). Taken together, because of the complex matter at hand, it is hard to determine whether microglia provide a net beneficial or detrimental role in AD, and a simple binary answer is not likely.

Despite the potential for global or spatially restricted alterations in transcriptome signature in aging and pathology, recent publications seem to suggest a common—or at least highly similar—transcriptional program (50, 51) in diseased states [i.e., disease-associated microglia (DAM) (52), microglia neurogenerative phenotype (53), ARM (40), and, most recently, white matter–associated microglia (54), a recently identified microglial phenotype that expands during aging and in neurodegenerative disease]. Massively parallel single-cell analysis with chromatin profiling on immune (CD45+) cells from 5XFAD mouse brains (a well-established AD mouse model) revealed two unique microglial clusters (52). Intriguingly, these clusters expressed genes implicated in lipid metabolism and phagocytosis (52). By analyzing mice from the age of 1-8 months (as Aβ deposition is advancing), the authors uncovered an age-dependent shift from a homeostatic phenotype to a DAM phenotype with one of the two clusters identified as being a transitory stage (defined as stage 1) (52). DAM are characterized by the up-regulation of *Itgax, Trem2, Axl, Cst7, Ctsl, Lpl, Cd9, Csf1, Ccl6, Clec7a, Lilrb4*, and *Timp2* (52). Notably, *Itgax* in particular was found in every cell featuring a DAM transcriptome signature (52). In addition, they down-regulate *Cx3cr1, P2ry12*, and *Tmem119* (52), genes typically expressed in homeostatic microglia (12). The authors further identified that DAM are in close association with Aβ plaques and contain phagocytosed Aβ (52). Intriguingly, the authors identified that a transition from stage 1 DAM to stage 2 DAM was dependent on triggering receptor expressed on myeloid cells 2 (*Trem2*) (52); homozygous loss-of-function mutations in this gene are known to cause autosomal recessive early-onset dementia [Nasu–Hakola disease and frontotemporal dementia (FTD)–like disease] (55–57). TREM2 acts as a receptor for apolipoprotein E, Aβ, and high- and low-density lipoprotein and has been identified as crucial for triggering microglial phagocytosis, proliferation, and inflammation (58). Importantly, loss-of-function mutations in *Trem2* have also been implicated in diverse neurodegenerative diseases including AD (59), amyotrophic lateral sclerosis (60), Parkinson disease (61), and FTD (62). Consistent with this, DAM transcriptomes have now been identified in diverse models of neurodegenerative disease. Based on these elegant findings, it is tempting to speculate that the DAM phenotype represents a pan-neurodegenerative disease response.

## Gradual Accumulation of Dam During Aging

Even though we and others have shown morphological and functional changes of microglial cells in aged mice, only a rather small subpopulation show a transcriptomic profile consistent with that of DAM. By comparing (Figure 1B) the top up-regulated genes (greater than 1.5-fold change) between DAM (52) and the small clusters of transcriptionally distinct cells from aged mice (OA2 and OA3) (12), a set of 39 mutually expressed genes can be identified between DAM and OA2 (Figure 1C). Conversely, only nine genes were mutually expressed between DAM and OA3 (Figure 1D). The amount of overlap in genes up-regulated in DAM and OA2 appears to support the hypothesis that a common transcriptional program is activated in both DAM and aged microglia, but only in a minority of the cell population in healthy aging. However, in various disease models, the cell population displaying transcriptomic signatures consistent with DAM is much larger, suggesting that pathological insults during the animal's life span will heavily expand the DAM population, which has a potential impact on cellular aging. Consistent with this, the ARM phenotype described by Frigerio et al. (consistent with DAM) eventually becomes the majority population of microglia at 12 months in an AD mouse model (*App*^*NL*−*G*−*F*^) (40). IRM (consistent with OA3) conversely seemed to increase with age more rapidly in *App*^*NL*−*G*−*F*^ mice than wild-type mice but ultimately also represented only a minority of cells (< ~5%) similar to wild-type mice. Ferretin expression has been identified as a marker for dystrophic microglia (36) and senescence (26). Shahidehpour and colleagues (36) found ferretin-expressing dystrophic cells to be present, but again to a very small extent, in healthy aged humans. Conversely, the number of dystrophic microglia was significantly increased in patients suffering from neurodegenerative disorders. Consistent with the data of Shahidehpour et al. (36), ferritin is expressed in the OA2 subpopulation (12), which again is minimal in the absence of overt pathology, yet abundant in neurodegenerative disease conditions (DAM) (52). Taken together, the data suggests a disease-induced increase of cellular aging hallmarks.

## Conclusions

Microglia with transcriptomic signatures consistent with that found in neurodegenerative diseases represent only a minority of microglia in healthy aging. It remains unclear what this subset of microglia contributes toward overall microglial dysfunction or, conversely, if they might have a beneficial impact. With regard to microglia featuring a neurodegenerative disease-associated transcriptome signature, it is possible that neurodegenerative disease causes advanced cellular aging, or conversely, advanced cellular aging may be a contributing factor to neurodegenerative disease (63). Further studies will be required to interrogate the roles of these interesting microglial populations in old age. In addition, it seems reasonable to speculate that data gathered from mice at the end of the mouse life span (~2.5 years) would be particularly valuable given the advanced ages reached by humans in contemporary society. scRNAseq studies to date seem to suggest that, despite the many factors that could potentially influence microglial phenotypes in either a global or spatially restricted manner, microglia in aged mice appear to consist of homeostatic microglia, neurodegenerative disease-like microglia, and IRM. The lack of apparent heterogeneity could conceivably be in part due to the artificial conditions that laboratory rodents reside in. This is an important caveat that should be considered when attempting to extrapolate observations from laboratory rodents to humans. With highly individualized lifestyles, disease backgrounds, and environmental factors, microglia in humans are likely to be primed more diversely and extensively given human longevity in comparison to laboratory mice. Hence, much remains to be discovered that could potentially bring valuable mechanistic insights into both aging and neurodegenerative disease.

## Author Contributions

MC and JKH wrote the manuscript. All authors contributed to the article and approved the submitted version.

## Conflict of Interest

The authors declare that the research was conducted in the absence of any commercial or financial relationships that could be construed as a potential conflict of interest.
